# Virus versus host: influenza A virus circumvents the immune responses

**DOI:** 10.3389/fmicb.2024.1394510

**Published:** 2024-05-16

**Authors:** Guanming Su, Yiqun Chen, Xiaowen Li, Jian-Wei Shao

**Affiliations:** ^1^College of Veterinary Medicine, South China Agricultural University, Guangzhou, China; ^2^Guangdong Provincial Key Laboratory of Veterinary Pharmaceutics Development and Safety Evaluation, Guangzhou, China; ^3^Key Laboratory of Zoonosis Prevention and Control of Guangdong Province, Guangzhou, China; ^4^School of Life Science and Engineering, Foshan University, Foshan, China

**Keywords:** influenza A virus, innate immunity, adaptive immunity, host-virus interaction, immune escape

## Abstract

Influenza A virus (IAV) is a highly contagious pathogen causing dreadful losses to humans and animals around the globe. As is known, immune escape is a strategy that benefits the proliferation of IAVs by antagonizing, blocking, and suppressing immune surveillance. The HA protein binds to the sialic acid (SA) receptor to enter the cytoplasm and initiate viral infection. The conserved components of the viral genome produced during replication, known as the pathogen-associated molecular patterns (PAMPs), are thought to be critical factors for the activation of effective innate immunity by triggering dependent signaling pathways after recognition by pattern recognition receptors (PRRs), followed by a cascade of adaptive immunity. Viral infection-induced immune responses establish an antiviral state in the host to effectively inhibit virus replication and enhance viral clearance. However, IAV has evolved multiple mechanisms that allow it to synthesize and transport viral components by “playing games” with the host. At its heart, this review will describe how host and viral factors interact to facilitate the viral evasion of host immune responses.

## Graphical abstract

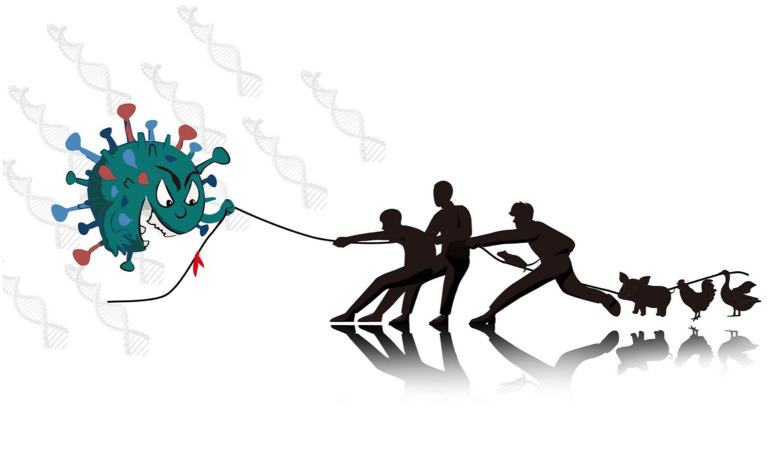

## 1 Introduction

Influenza A virus (IAV), a negative-stranded RNA virus containing eight single-stranded RNA segments, belongs to the *orthomyxoviridae* family and can infect birds, humans, and other mammals. Indeed, IAV infection has become a global risk factor (Richard and Fouchier, 2016), with outbreaks that can cause up to 5 million severe illnesses and up to 500,000 deaths worldwide (Gopal et al., 2020).

The host’s innate immunity is the initial line of defense against viral invasion, especially the interferon (IFN) response (Tan et al., 2018). Despite the potent antiviral activity of the innate immune system, it is interesting that viruses, to escape from strong immune responses, have evolved tactics to subvert the host immune repertoire and thereby establish successful infection and proliferation. Antigenic drift, a minor change in the antigenicity of IAV caused by site mutations of surface glycoprotein (either hemagglutinin or neuraminidase), can lead to annual seasonal epidemics (Webby and Webster, 2001), while antigenic shift is to blame the HA or NA recombination events and can generally cause a virus pandemic (Gao et al., 2013). Even healthy young populations vaccinated in advance may have little or no protective immunity when infected with IAV, which significantly increases the mortality rate.

Presently, the effects of immune escape are intricate and multi-dimensional, and myriad outstanding achievements have been accumulated in existing studies. However, the in-depth immune escape study needs a recent comprehensive content summary highlighting the importance of assessing IAV infection and the immune responses to control and eliminate the infection. Therefore, this review summarizes the latest research on how IAVs evade the host immune system and gain the upper hand in the virus-host game.

Most importantly, this review aims to answer the following research questions: How does IAV elude immune defense? What viral proteins and host factors are involved in this process? On the other hand, it allows researchers to put some order into the current patchwork of studies on immune evasion strategies utilized by IAV. Finally, we discuss some major gaps and unknowns on this subject.

## 2 Morphological structure and protein function of IAV

**Figure 1:**
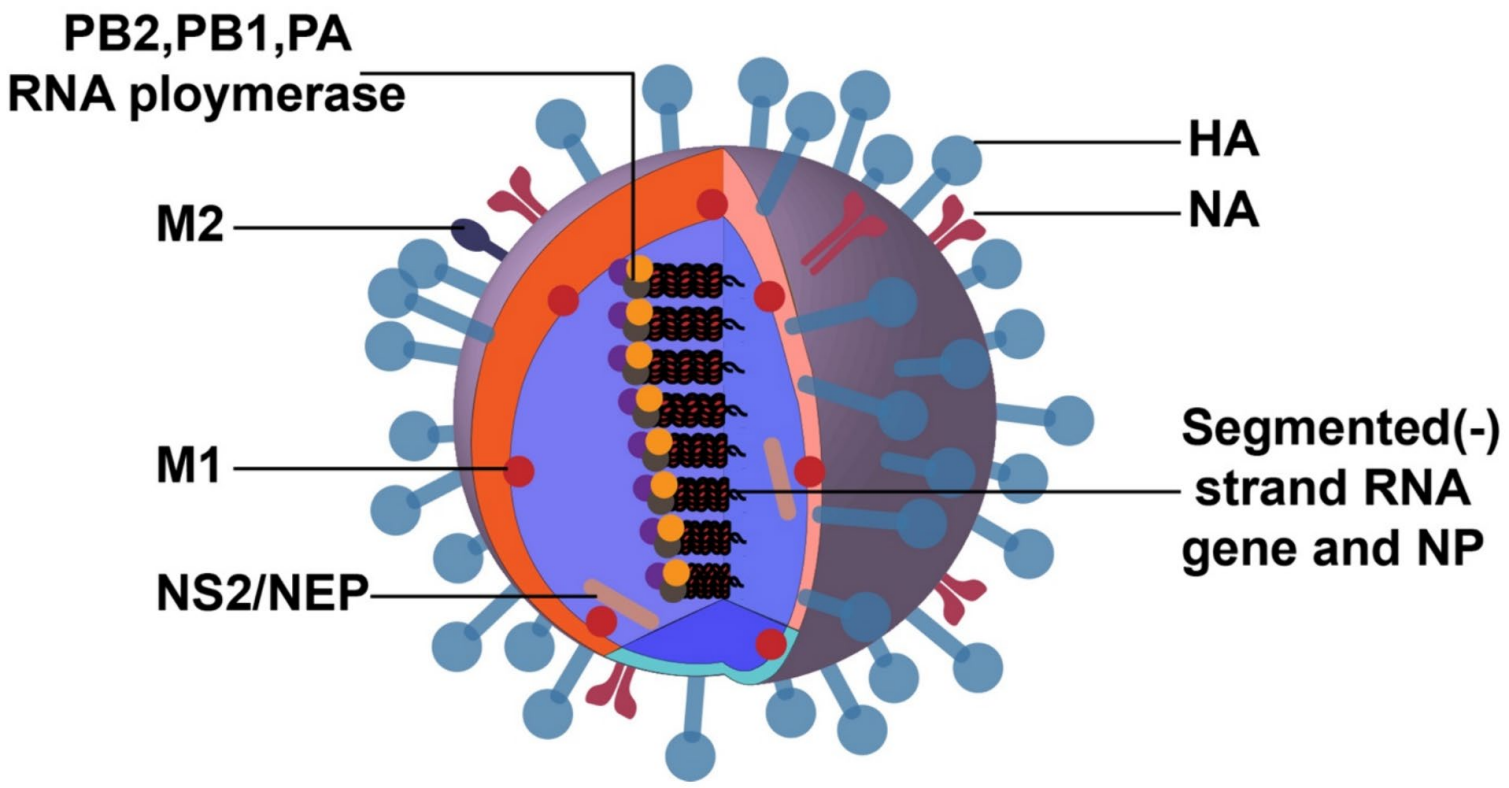
Structure of IAV. The image was created with Adobe Illustrator.

IAV has a broad distribution spectrum, and its main host is waterfowl (Olsen et al., 2006). The viral RNA (vRNA) genome is comprised of eight negative-sense RNA segments (Figure 1). The core proteins encoded by vRNA are polymerase basic 2 protein (PB2), polymerase basic 1 protein (PB1), polymerase acidic protein (PA), hemagglutinin protein (HA), nucleoprotein (NP), neuraminidase protein (NA) and matrix proteins (M1 and M2), nonstructural proteins (NS1 and NS2).

The shape of virus particles is not static, canonically spherical, and a few are filamentous. The surface glycoproteins of virions include HA and NA, and their antigenicity can be used to distinguish and name different virus subtypes. The HA spikes have receptor binding and fusion functions, and NA spikes have receptor-destroying activity. The envelope also contains a third integral membrane protein, M2, which is exposed on the outer surface and functions as an ion channel, essential for uncoating. There are 18 HA subtypes, and 11 NA subtypes unlocked in nature (Yang et al., 2021; Fereidouni et al., 2023). Among these subtypes, there are 3 HA (H1, H2, and H3) and 2 NA (N1 and N2) subtypes of IAV that have occasionally infected humans and caused annual epidemics (Watanabe et al., 2014).

The density and thickness of the M1 protein align with the host cell capsule layer. M1 associates with viral ribonucleoprotein (vRNP) and the virion core and connects with the envelope glycoproteins, HA and NA, to support the virus particle structurally (Schaap and Eghiaian, 2012). The innermost component of the virion is the vRNP complex, composed of vRNA, polymerase proteins, and NP (Moeller et al., 2012). It is characterized by being imported into the cytoplasm for virus assembly and budding at the late stage of the IAV life cycle.

## 3 IAV escapes from innate immune

### 3.1 Viral proteins mediate immune escape

The initial response to IAV is directed by the innate immune system, which is triggered by the host’s PRRs recognizing viral PAMPs. PRRs are composed of membrane-bound Toll-like receptors (TLRs), cytoplasmic bound retinoic acid inducer I (RIG-I) -like receptors (RLRs), and nucleotide-binding oligomerization domain (Nod) -like receptors (NLRs) (Zhang et al., 2022), mediate viral sensing and activate downstream transcription factors and interferons (IFNs). IFNs initiate immune responses in infected and neighboring cells. The innate immune response releases mounting IFN-stimulated genes (ISGs), leading to the arrest of viral replication (Negishi et al., 2018; Chen et al., 2021).

IAV has evolved multiple strategies to circumvent host immunity to establish successful infection. For example, IAV infection inhibits IFN signaling to reduce IFN production, thus suppressing many antiviral molecules’ expression. The suppression of innate immunity by AIV is a sophisticated mechanism and mainly involves a variety of viral proteins (PB1, PB2, PA, NS1, and PB1-F2).

#### 3.1.1 NS1 protein

**Figure 2:**
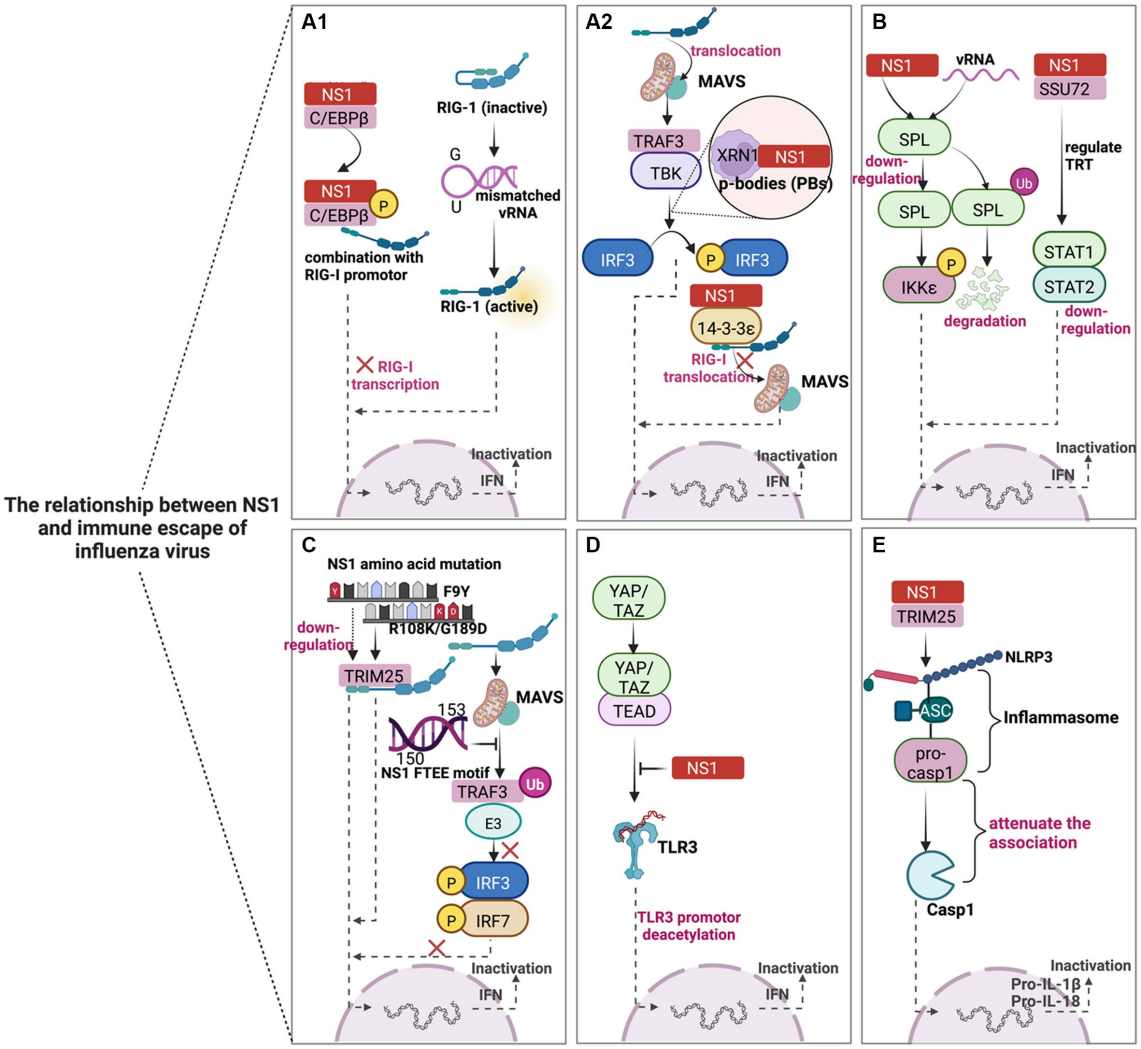
Schematic diagram of the mechanism of NS1-mediated immune escape in IAV. NS1 mediates the innate immune escape of viruses in five main ways. **(A–D)** could reduce IFN-β synthesis by inhibiting IFN signaling pathways, thereby circumventing the antiviral effect of host immunity, while E contributes to the immune escape of IAV by inhibiting the inflammatory response pathway. **(A1,A2)** mainly play the virus against the host by blocking the RIG-I signaling pathway. **(B)** NS1 mainly acts through host factors (SPL and SSU72) to subvert the antiviral response. **(C)** The NS1 protein can escape host innate immunity through amino acid substitutions such as single mutation F9Y, double mutation (R108K/G189D), and conserved FTEE motif (aa150-153). **(D)** NS1 truncation leads to the blocking of TLR production and weakens the antiviral effect. **(E)** NS1 can also interact with TRIM25 through the NLRP3-mediated inflammasome activation pathway to reduce IL-1β signaling, which may have additional antagonistic effects on host pro-inflammatory responses and create a favorable environment for viral replication. The image was created with BioRender.com.

Non-structural protein 1 (NS1) is considered a pivotal fraction in enhancing virulence by antagonizing the innate antiviral response in various hosts (Figure 2). As stated above, the RIG-I, TLR3, and TLR7 can detect viral RNA in different cell types to induce type I IFN antiviral response upon IAV infection.

NS1 inhibits the activity of RIG-I mainly through direct or indirect influences, enabling the virus to counteract the host antiviral innate immune response successfully (Figures 2A1,A2). For instance, overexpression of CCAAT/enhancer binding protein beta (C/EBPβ) leads to the inhibition of RIG-I expression, while NS1 can promote the phosphorylation process of C/EBPβ and C/EBPβ/NS1 complex occupies the RIG-I promoter, which ultimately promotes viral replication in human lung epithelial cells (Kumari et al., 2020). The pan-handle structure of the vRNA promoter is reckoned to be sensed by RIG-I; the results demonstrated that the mismatched vRNA promoter could affect the formation of the RNA/RIG-I complex and prevent RIG -I activation. In other words, mismatch may represent a general viral strategy to escape RIG-I sensing (Anchisi et al., 2016). IAV can also hijack cellular 5′-3′ exoribonuclease 1 (XRN1) to increase viral titers. IAV NS1 directly interacts with cellular XRN1 and co-localizes in the P-bodies (PBs), and the virus utilizes XRN1 activity to inhibit RIG-I mediated innate immune response (Liu et al., 2021). Additionally, IAV NS1 competitively binds to 14–3-3ε protein with RIG-I to inhibit the translocation of RIG-1 to the adaptor MAVS, leading to insufficient IFN-β production (Wang et al., 2022).

Studies have shown that sphingosine 1-phosphate (S1P) lyase (SPL) enhances the IKKε-mediated type I IFN response, and IAV infection or NS1 protein expression can cause ubiquitination or down-regulation of SPL. Thus, the SPL-mediated antiviral response is subverted (Figure 2B) (Wolf et al., 2021). However, many questions remain unanswered regarding the underlying mechanism of NS1 triggering SPL ubiquitination and degradation. Compared with less virulent seasonal IAV, highly pathogenic avian influenza virus (HPAIV) H5N1, H5N6, and H7N9 can better bypass the innate immune response. Furthermore, through the molecular mechanism of the NS1-SSU72-trans-transcriptional readthrough (TRT)-STAT1/2 axis, AIV infection can reduce the expression of SSU72 and induce TRT, thereby weakening the innate immune response (Figure 2B) (Zhao et al., 2022). Thus, restoring SSU72 expression could be a potential strategy for preventing AIV pandemics.

The NS1 protein can escape innate immunity through amino acid substitution (Figure 2C). The NS1 F9Y amino acid mutation of H1N1 introduced by error-prone PCR can significantly enhance virus growth *in vitro* and *in vivo*, increasing virus virulence in mice (Yu et al., 2022). This characteristic mutation can target antiviral drugs and attenuate vaccine development. In addition, the double mutation (R108K/G189D) in the NS1 protein suggested a systematic and selective inhibition of cytokine responses to counteract the innate immune response (Huang et al., 2021). At the same time, the conserved FTEE motif (aa150-153) of NS1 can subvert the RIG-I, TLR3, and TLR7 pathways in combination with TRAF3 E3 ubiquitin ligase to produce type I IFN (Lin et al., 2021). Therefore, the “power” of the virus to antagonize host immunity should not be underestimated by the changes in crucial amino acids.

TLR and NLR significantly contribute to innate immunity against the IAV. The hippo signaling pathway can act as an immune regulator. NS1 binds to the C-terminal domain of the hippo effectors (Yes-associated protein (YAP) and transcriptional coactivator with PDZ-binding motif (TAZ)) YAP/TAZ complex. It activates YAP/TAZ, hijacking the complex into the nucleus. At the same time, YAP/TAZ can downregulate the expression of TLR3 and ultimately block the antiviral innate immune signal, facilitating viral replication and host cell apoptosis (Figure 2D) (Zhang et al., 2022). In addition, NS1 can also interact with tripartite motif-containing protein 25 (TRIM25) through the NLRP3-mediated inflammasome activation pathway. NS1 attenuates the association between pro-caspase-1 and ASC, followed by decreasing IL-1β signal transduction. Collectively, NS1 has an additional antagonistic effect on proinflammatory response to create a favorable environment for viral replication (Figure 2E) (Park et al., 2021).

#### 3.1.2 Polymerase proteins

The PB2 protein of RNA polymerase utilizes another key inhibitory mechanism to mediate viral immune evasion. However, the PB2 protein can be localized in the nucleus and mitochondria, and the interaction between PB2 and MAVS can inhibit MAVS mediate IFN-β expression. Still, the PB2 protein of AIV has not been found to have such a phenomenon (Graef et al., 2010).

**Figure 3:**
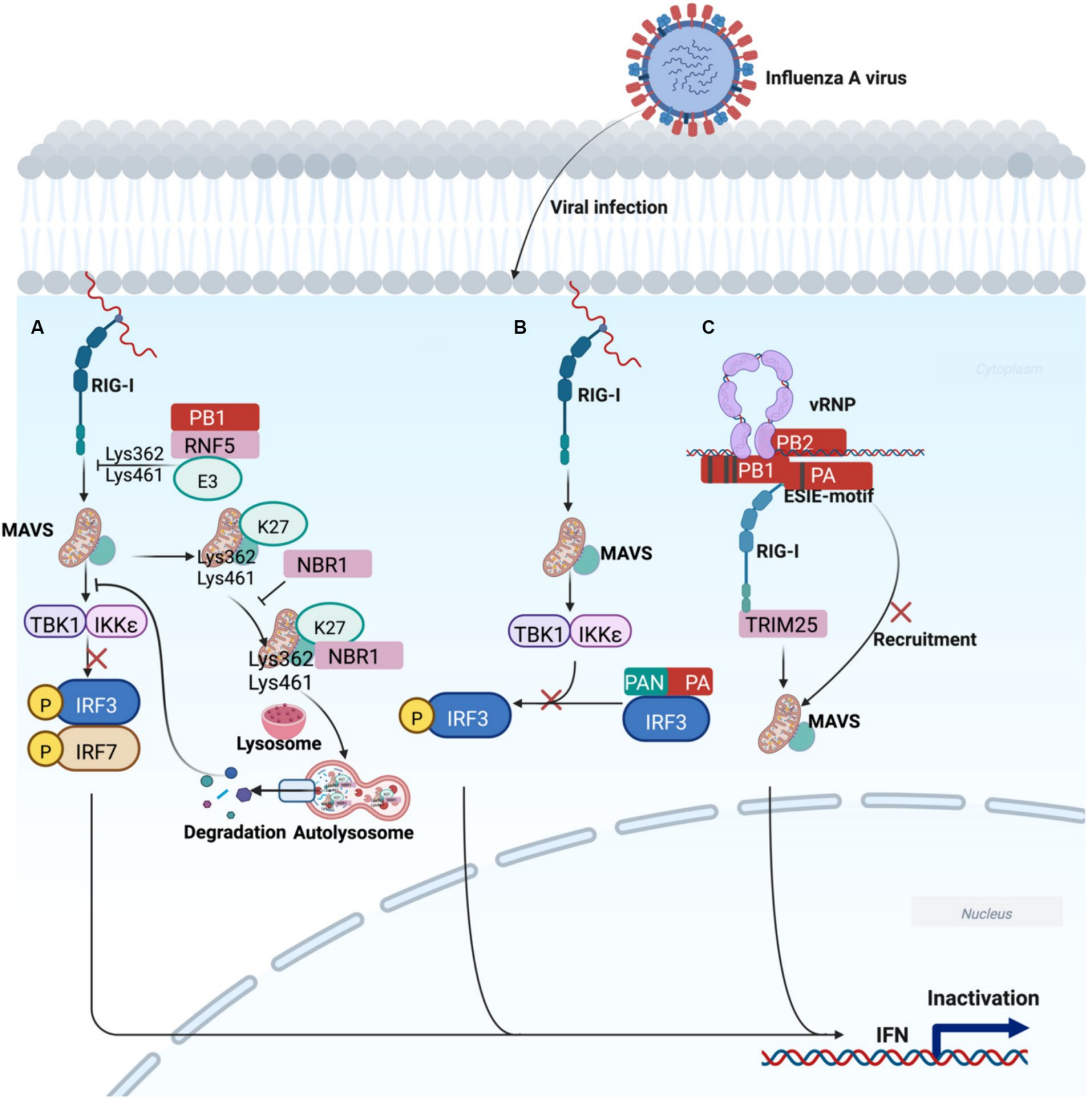
Working model of polymerase acidic proteins-mediated regulation of innate immune escape signal transduction. **(A)** PB1 delivers MAVS to autophagosomes for degradation through the PB1-RNF5-MAVS-NBR1 axis, blocking RIG-I-MAVS-mediated degradation, ultimately leading to IFN decline and promotion of H7N9 infection. **(B)** The N-terminal functional domain of PA blocks IRF3 phosphorylation, which is responsible for IFN-β suppression. **(C)** The ESIE motif can sufficiently antagonize the induction of IFN, which may be associated with the pandemic risk of IAV. The image was created with BioRender.com.

PB1 preferentially interacts with a selective autophagic receptor neighbor of BRCA1 (NBR1), which delivers MAVS to autophagosomes for degradation via the PB1-RNF5-MAVS-NBR1 axis, blocking RIG-I-MAVS-mediated innate signaling pathway to promote H7N9 infection (Figure 3A) (Zeng et al., 2021). In addition, PA can inhibit the IFN-β by interacting with IRF3, an alternative strategy IAV utilizes to antagonize the host immune response (Figure 3B) (Yi et al., 2017). Interestingly, PB1 (398E/524S/563I) and PA (351E)-referred to as ESIE-motif-adequately antagonized IFN expression, which leads to a failure to sense the virus, suggesting the combination of ESIE-motif with viral rearrangements may contribute to pandemic risk (Figure 3C) (Liedmann et al., 2014). However, the mechanism of IFN-β inhibition by the viral polymerase proteins is poorly understood.

#### 3.1.3 Novel proteins

**Figure 4:**
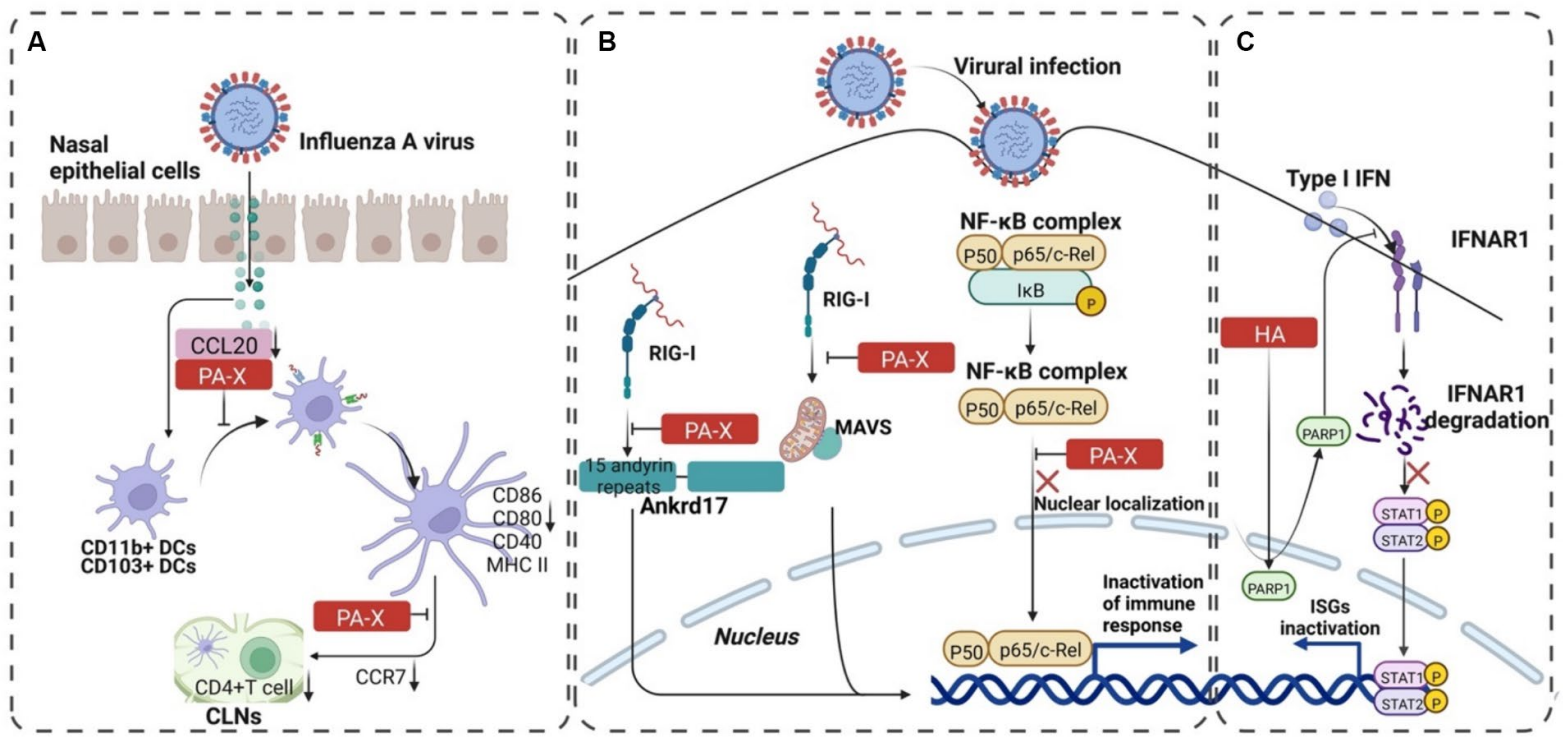
The progression of PA-X and HA-mediated immune escape of IAV. **(A)** PA-X protein assisted H9N2 subtype AIVs in escaping immune response of mucosal DCs. The binding of PA-X to CCL20 blocks the recruitment of CD11b^+^ CDs and CD103^+^ CDs to the nasal mucosa. It downregulates the expression of CCR7 to reduce the expression of CD4^+^ T cells, which mediates the adaptive immune escape of the virus. **(B)** PA-X reduces the expression of interferon by inhibiting RIG-I and NF-κB pathways and increases the replication advantage of the virus. **(C)** The interaction between HA and PARP1 decomposes IFNAR1, inhibits the phosphorylation of STAT1, and then reduces IFN-β, which finally mediates the innate immune escape of the virus. The image was created with BioRender.com.

PA-X, an overlapping protein-coding region in PA, encodes the N-terminal 191 amino acids and unique C-terminal sequences (41 or 61 amino acid residues) of the PA protein produced by +1 reading frame of the PA gene via ribosomal frameshifting (Jagger et al., 2012). PA-X protein inhibits the expression of chemokine CCL20 to reduce the recruitment of DCs to the nasal epithelial cells (Ecs), which assists the immune evasion of H9N2 subtype AIVs by blocking the downstream innate and mucosal immune activation (Figure 4A) (Qin et al., 2022). Another result showed that PA-X protein inhibited viral replication in chBM-DCs (chicken bone marrow-derived dendritic cells) but not in chicken embryo fibroblast cells (DF-1). Moreover, the PA-X protein downregulated the expression of phenotypic markers (CD40, CD86, and MHC II) and proinflammatory cytokine (IL-12 and IL-1β) of chBM-DCs. Taken together, these findings indicated that PA-X protein is a key viral protein that helps H9N2 subtype AIVs escape the innate immunity of chBM-DCs (Qin et al., 2023). In addition, PA-X acts as a positive viral replication regulator and interacts with host proteins to inhibit IFN expression (Figure 4B) (Rigby et al., 2019; Hu et al., 2020; Li et al., 2021).

PB1-F2 is a 90 amino acid protein expressed from the +1 open reading frame in the PB1 gene of IAV (Chen et al., 2001). Recently, it has been reported that PB1-F2 transport to mitochondria by interacting with the Tu translation elongation factor, mitochondria (TUFM), which in turn promotes complete mitophagy. As a result, MAVS is delivered to autophagosomes for degradation, and the type I IFN induction is then suppressed (Wang et al., 2021). Albeit duck and human MAVS share only 28% amino acid similarity, PB1-F2 inhibits both human and avian MAVS signaling to suppress IFN-β induction (Xiao et al., 2020). Of course, there are some exceptions. For example, PB1-F2 of the H7N9 AIV can significantly induce mitochondrial ROS production, accelerate the activation of NLRP3, and negatively regulate virus replication (Pinar et al., 2017).

#### 3.1.4 HA protein

The IAV employs a sophisticated strategy to evade the host’s innate immune response, with the HA protein playing a pivotal role. Our investigation sheds light on the intricate mechanisms underlying HA-mediated immune evasion:

IFNAR1 (IFN-alpha receptor 1) Degradation by HA1 Protein:

- The HA1 subunit of the HA protein orchestrates IFNAR1 ubiquitination, leading to a reduction in IFNAR1 levels within infected cells (Xia et al., 2015).
- Remarkably, the abundance of IFNAR1 on the cell surface directly influences cellular sensitivity to interferon-alpha/beta (IFN-α/β) signaling (Kumar et al., 2004, 2007; Zheng et al., 2011).
- A comprehensive study delved into the mechanism of IFNAR1 degradation by IAV HA. Intriguingly, HA not only targets IFNAR1 but also degrades the type II interferon (IFN-γ) receptor 1 (IFNGR1) via casein kinase 1α (CK1α). Knockdown of CK1α using small interfering RNA (siRNA) effectively repressed the degradation of both IFNAR1 and IFNGR1 induced by IAV infection (Xia et al., 2015, 2018).

Role of PARP1 [Poly (ADP-ribose) polymerase 1] in IFN Receptor Degradation:

- Mass spectrometry (MS) studies unveiled the molecular mechanism underlying IFN receptor degradation by IAV HA.
- Cellular PARP1 emerges as a critical mediator of IAV HA-induced IFNAR1 degradation.
- IAV HA dynamically alters the distribution of endogenous PARP1, facilitating the orchestrated degradation of IFNAR1. This process significantly benefits IAV propagation (Figure 4C) (Xia et al., 2020).

Uncharted Territory: HA and Innate Immune Escape:

- Despite these intriguing findings, our understanding of HA’s role in innate immune escape remains incomplete.
- Further exploration is warranted to unravel the precise mechanisms by which HA orchestrates immune evasion, especially considering its potential impact on pandemic risk.

#### 3.1.5 NP protein

Autophagy is an important cellular process to maintain cellular homeostasis by cleaning up the hazardous substrates in lysosomes. Accumulating research has also suggested that autophagy is a critical mechanism in host defense responses against IAV infection by degrading viral particles and activating innate or acquired immunity to induce viral clearance. However, IAV has conversely hijacked autophagy to strengthen virus infection by blocking autophagy maturation and further interfering with host antiviral signaling to promote viral replication (Krammer et al., 2018). A study revealed that NP-mediated mitophagy leads to the degradation of the mitochondria-anchored protein MAVS, thereby blocking MAVS-mediated antiviral signaling and promoting virus replication. The NP-mediated mitophagy depends on the NP’s interaction with MAVS and the cargo receptor TOLLIP (toll-interacting protein). Moreover, Y313 of NP is a key residue for the MAVS-NP interaction and NP-mediated mitophagy. The NPY313F mutation significantly attenuates the virus-induced mitophagy and the virus replication *in vitro* and *in vivo* (Zhang et al., 2023).

### 3.2 Host factors mediate immune escape

#### 3.2.1 Host proteins

IAV utilizes host factors to inhibit the expression of IFNs for efficient transmission, which is the most common way of immune escape (Table 1). N-Myc and STAT (signal transducer and activator of transcription) interactor (NMI) is an IFN-induced protein. Upon IAV infection, NMI recruits the TRIM21 (tripartite motif-containing 21, an E3 ubiquitin ligase) to IRF7 and promotes proteasomal degradation of IRF7. Consequently, NMI impairs the IRF7-induced type I IFN response and facilitates IAV infection (Ouyang et al., 2021). IAV manipulates Src homology region 2-containing protein tyrosine phosphatase 2 (SHP2) to activate the epidermal growth factor receptor (EGFR) signaling pathway, which leads to the downregulation of IFNs and suppression of innate antiviral immunity (Wang et al., 2021). IAV infection upregulates the suppressor of cytokine signaling (SOCS) protein SOCS1, which antagonizes the IFN-activated JAK/STAT signaling pathway by inducing the degradation of JAK1, resulting in suppressed type I and type II IFN responses (Du et al., 2020). Sphingosine kinase 2 (SPHK2), a host sphingolipid metabolism-related factor, interacts with IFN-β promoter through the binding of demethylase TET3 but not with the other promoters regulated by TET3, can in turn recruit HDAC1 to the IFN-β promoter, enhancing the deacetylation of IFN-β promoter, therefore negatively regulating type I IFN expression and thus facilitates IAV propagation (Xu et al., 2022). From RNA interference (RNAi) screening, the double plant homeodomain fingers 2 (DPF2/REQ) gene was identified as one of six genes for which knockdown significantly decreased the infectivity of IAV. DPF2 negatively regulated the induction of the type I IFN and strongly suppressed activation of the JAK/STAT signaling pathway in IAV-infected cells in response to promoting the immune escape of IAV (Shin et al., 2017). As such, PR8 infection specifically induces transcription factor RUNX1, which blocks the expression of IRF3 and STAT1 to attenuate the production of IFN-β and ISGs to facilitate viral infection in A549 cells (Hu et al., 2022). The RNase activity of MCP-1-induced protein 1 (MCPIP1) can directly degrade cytokine mRNAs, such as IL-6, IL-12, IL-1β, and IL-2, by functioning as an RNase. The study showed that MCPIP1 reduced RIG-I expression after IAV-infected A549 cells by disrupting the stability of RIG-I mRNA, thereby diminishing the IAV-induced host antiviral response (Sun et al., 2018).

**Table 1:** Summary of host factors involved in innate immune escape in IAV infection.

Virus	Host proteins	Pathway	References
H1N1	NMI	Viral infection-NMI↑-Recruits TRIM21/E3-IRF7^Ub^/K48↓-Type I IFN↓	Ouyang et al. (2021)
H1N1	SHP2	Viral infection-SHP2↑-EGFR/ERK^ppp^↑-IFN↓-ISG↓	Wang et al. (2021)
H5N1/H7N9/H9N2	SOCS1	Viral infection-SOCS1↑-IFN-α/γ↓- Degradation of JAK1-STAT1^ppp^↓	Du et al. (2020)
H1N1	SPHK2	Viral infection-SPHK2 nuclear accumulation↑-SPHK2 translocation↑-Recruits TET3 and HDAC1↑-Deacetylation of IFN-β promoter-IFN-β↓	Xu et al. (2022)
H1N1/H3N2	DPF2	Viral infection-DPF2↑-IFN-β↓-JAK/STAT-STAT1^ppp^↓-MxA, ISG56, IL-8, IP-10, and IL-6↓	Shin et al. (2017)
H1N1	RUNX1	Viral infection-RUNX1↑-IRF3^ppp^↓-IFN-β↓-STAT1^ppp^↓-MxA/ISG15↓	Hu et al. (2022)
H1N1	MCPIP1	Viral infection-MCPIP1 (RNase activity) ↑-RIG-I mRNA↓-IFN-β↓	Sun et al. (2018)
HPAIV H5N1	NDRG1	Viral infection-NDRG1↑-Targets IKKβ-IFN-β and IL-8↓	Chen et al. (2018)
H3N2	Sox4	(1) Viral infection-Sox4↑-IKKα/β^ppp^↓-Inhibition of NF-κB activity and protein degradation of IRF3/7	Shang et al. (2021)
		(2) Viral infection-Sox4↑-Blocks the activities of the TLR/MyD88/IRAK4/TAK1 or TLR/TRIF/TRAF3/TBK1 pathways-IFN↓-ISG↓	
H3N2	NOX2	Viral infection-NOX2↑-H_2_O_2_↑-Modifies cysteine residue of TLR7 (Cys98)-IFN-β, IL-1β, TNF-α and IL-6↓	To et al.(2017)
H1N1	PSMA2 NRF2	Viral infection-PSMA2↑- NRF2 accumulation in the nucleus-activates expression of antioxidant HO-1 proteins-ROS↓	Rashid et al. (2022)
H1N1	Focal adhesion kinase (FAK)	IAV hijacks FAK-Inhibition of FAK activity-Inhibition of NF-κB activity-Inhibition of NF-κB nuclear translocation	Bergmann and Elbahesh (2019)
H1N1	Guanylate-binding protein 7 (GBP7)	(1) Viral infection-GBP7↑-IKKα^ppp^↓-Inhibition of NF-κB activity-Inhibition of nuclear translocation	Feng et al. (2021)
		(2) Viral infection-GBP7↑-IFN↓-STAT1^ppp^/STAT2^ppp^↓-ISG↓	

IAV relies on positive cytokines and decreases the activity of NF-κB-related signaling pathways, which is one of the common ways of immune escape. The transcriptional repressor N-myc downstream-regulated gene 1 (NDRG1) facilitates IAV replication by targeting IKKβ and promotes HPAIV H5N1 replication by inhibiting canonical NF-κB activity (Chen et al., 2018). In addition, the sex-determining region Y-box 4 (Sox4) is induced to be up-regulated during IAV infection. Sox4 inhibits the production of MyD88 and most TLRs by binding to their promoters to attenuate gene transcription. In addition, Sox4 blocks the activities of the TLR/MyD88/IRAK4/TAK1 and TLR/TRIF/TRAF3/TBK1 pathways by repressing their key components. Moreover, Sox4 represses the activation of the NF-κB by interacting with IKKα/β and attenuates NF-kB and IRF3/7 abundances by promoting protein degradation. All these contributed to the down-regulation of IFNs and ISG expression, facilitating viral replications and shutting down host immunity to assist IAV (Shang et al., 2021). Notably, the major enzymatic source of ROS, NOX2 oxidase, is activated by a variety of ssRNA and DNA viruses, including IAV, in endocytic compartments, followed by the production of endosomal hydrogen peroxide (H_2_O_2_), which modifies a unique and highly conserved the cysteine residue (Cys98) of TLR7 to promotes viral replication (To et al., 2017). In addition, proteasome subunit alpha type 2 (PSMA2) is a cellular protein highly expressed in IAV-infected human lung epithelial A549 cells. The result showed that PSMA2 was required for nuclear factor erythroid-2-related factor 2 (NRF2)-mediated ROS neutralization and that IAV uses PSMA2 to escape viral clearance via the NRF2-mediated cellular oxidative response (Rashid et al., 2022). Nevertheless, the ROS-dependent regulation of antiviral immunity remains a direction worthy of further exploration.

#### 3.2.2 Non-coding RNAs

IAV can circumvent the host’s innate immune system by regulating certain host-long non-coding RNAs (lncRNAs) for their effective propagation (Li et al., 2019; Wang and Cen, 2020). LncNSPL directly binds to RIG-I and blocks the interaction between RIG-I and TRIM25 by reducing TRIM25-mediated lysine 63 (K63)-linked ubiquitination of RIG-I, inhibiting IFN-β activation and reducing the production of antiviral cytokines (Jiang et al., 2022), which is similar to the mechanism mediated by the interaction between NS1 and TRIM25 (Gack et al., 2009).

Increasing evidence has shown that IAV can alter the expression profile of host miRNA (Zhang et al., 2019). Some host miRNAs are involved in various viral infections by regulating type I IFN (Ho et al., 2014). *In vitro*, miR-221 down-regulation promotes IAV replication by targeting the SOCS1/NF-κB pathway to inhibit type I IFN response (Zhang et al., 2021). Besides, miR-194 can directly target fibroblast growth factor 2 (FGF2), down-regulating FGF2 expression at protein transcription and translation levels to limit IFN response and accelerate H5N1 virus replication (Shi et al., 2020).

## 4 IAV escapes from adaptive immune

Host recognition of IAV invasion stimulates innate immune signaling, which leads to the synthesis of numerous effectors and directs the adaptive immune response to the corresponding type of response (Iwasaki and Medzhitov, 2015). Adaptive immunity is the second line of defense against pathogenic microorganisms, including humoral and cellular immunity.

In humoral immunity, the host mainly produces neutralizing antibodies against HA protein. The antibodies bind to HA spherical heads on the virions, inhibiting the attachment of IAV to host cells. In contrast, binding of HA on the surface of infected host cells is required to initiate antibody-dependent cell-mediated cytotoxic effects (ADCC) to eliminate IAV (de Jong et al., 2000). However, antibodies produced against the NA protein have no neutralizing activity, and their major effect is to inhibit NA’s enzymatic activity, thereby preventing the release of progeny virions from infected cells (Krammer, 2019).

Cellular immunity is dominated by memory T cells. IAV antigens are presented to CD4^+^ T cells via major histocompatibility complex (MHC) class II molecules and subsequently activated to produce T helper (Th1) and Th2 cells. Th1 cells express IFN gamma (IFN-γ) and IL-2, which promote the proliferation and differentiation of cytotoxic T lymphocytes (CTLs). Th2 cells express IL-4, IL-5, and IL-13, activating B cells’ differentiation and producing specific antibodies. In acquired immunity, DCs are involved in innate immunity and crucial antigen-presenting cells (APCs). DCs present endogenous antigens to CD8^+^ T cells via MHC class I molecules, capable of activating and differentiating into IAV-specific CTLs. CTLs then migrate to the site of infection, recognizing and eliminating cells infected with multiple IAV subtypes (Ekiert et al., 2011).

Clearance of viral infection by adaptive immunity is mediated by neutralizing antibodies produced by humoral immunity and immune cells produced by cellular immunity. The body produces Th1 immunity to clear the IAV from the lungs (Graham et al., 1994). DCs initiate primary immune responses after recognizing pathogens (Mellman and Steinman, 2001), present antigens to MHC II and MHC I, and up-regulate the expression of costimulatory molecules, which are eventually processed by CD4^+^ T cells and cytotoxic CD8^+^ T cells that lyse virus-infected cells and secrete IFN-γ to initiate Th1 immunity (Moran et al., 1999).

Many studies have demonstrated that the substitution of HA protein amino acids is a key determinant of viral antigenic variation (Kaverin et al., 2004; Okamatsu et al., 2008; Ping et al., 2008; Wan et al., 2014; Zhu et al., 2015, 2022; Peacock et al., 2016; Jin et al., 2019; Xu et al., 2022; Lyashko et al., 2024), and the antigenic drift of NA may be a by-product of HA (Hensley et al., 2011). The two have co-evolved to assist IAV immune escape. The antigenicity changes of H9N2, H7N9, and H5NX subtype AIVs are pushed by the renewal of vaccine strains and the pressure of antibody selection. Viral evolution promotes viral escape from the neutralization of antibodies by adding N-linked glycosylation (NLG) to shield the antigenic sites, changing virus-antibody binding, or altering the specificity of receptor binding. Adding glycosylation sites at the mutated amino acid sites carried in proteins HA and NA can induce virion escape (Chang and Zaia, 2019; Sealy et al., 2020; Bao et al., 2021; Ge and Ross, 2021; Thornlow et al., 2021). Still, glycosylation is often accompanied by the emergence of compensatory mutations whose function is unknown. Obviously, the exact mechanism of how IAVs escape from adaptive immunity remains to be elucidated (Kosik et al., 2018).

## 5 Conclusion and future perspectives

In general, the latest research results on the IAV escaping the host’s innate immunity are abundant. We know that IAV uses its proteins and hijacks host proteins as the main means of escape. Recently, various novel viral proteins have been discovered to facilitate virus infection. Still, more studies are needed to explore the roles of these proteins in the IAV infection process and the detailed mechanisms of viral replication and successful antagonism against the host. Moreover, lncRNAs can mediate the antiviral response manipulated by host immunity (Chen et al., 2021; Yao et al., 2021). Hence, future studies should emphasize how viruses use host lncRNAs for immune escape.

A study identified that the dimerization domain of the SARS-CoV-2 nucleocapsid protein (SARS2-NP) is required for SARS2-NP to undergo liquid–liquid phase separation (LLPS) with RNA, which inhibits Lys63-linked poly-ubiquitination and aggregation of MAVS and thereby suppresses the innate antiviral immune response. Mice infected with an RNA virus carrying SARS2-NP exhibited reduced innate immunity, an increased viral load, and high morbidity (Wang et al., 2021). Future research can explore whether there is a relationship between liquid–liquid phase separation and the immune escape of the IAV.

IAV escaping from acquired immunity is a key “blind area.” Viral fitness requires compatibility between HA and NA. So, the IAV needs to change antigenicity so that antibodies cannot neutralize the IAV. Moreover, N-glycosylation of IAV is required for proper viral binding and release (Wagner et al., 2000; Daniels et al., 2003). The glycosylation of HA and NA can interfere with vaccine-induced antibodies by forming steric hindrance (Wagner et al., 2000; Chang and Zaia, 2019), ultimately leading to immune escape of IAV (Hervé et al., 2015). Data suggested that aberrant cellular glycosylation may increase the risk of severe influenza due to the increased ability of glycome-modified IAVs to evade the immune response. Specifically, immunization of mice with NGI-1-treated virus significantly reduced antihemagglutinin and antineuraminidase titers of total serum antibody and reduced hemagglutinin protective antibody responses (Alymova et al., 2022). However, the detailed escape mechanism has yet to be discovered. Indeed, the selection of model viruses is one of the difficulties in studying the immune escape of IAVs. Future studies should explore which adaptive immune signaling pathways are responsible for the failure of antibody neutralization. Only with a better understanding of how IAVs escape from the host immune response can we more effectively develop vaccines and antiviral drugs to achieve the purpose of precise prevention and control of influenza epidemics.

## Author contributions

GS: Writing – original draft, Writing – review & editing, Investigation. YC: Writing – original draft, Writing – review & editing, Investigation. XL: Writing – original draft, Writing – review & editing, Investigation. J-WS: Writing – original draft, Writing – review & editing.

## References

[ref1] Alymova I. V. Cipollo J. F. Parsons L. M. Music N. Kamal R. P. Tzeng W. P.. (2022). Aberrant cellular glycosylation may increase the ability of influenza viruses to escape host immune responses through modification of the viral Glycome. MBio 13, e02983–e02921. doi: 10.1128/mbio.02983-21 35285699 PMC9040841

[ref2] Anchisi S. Guerra J. Mottet-Osman G. Garcin D. (2016). Mismatches in the influenza a virus RNA panhandle prevent retinoic acid-inducible gene I (RIG-I) sensing by impairing RNA/RIG-I complex formation. J. Virol. 90, 586–590. doi: 10.1128/JVI.01671-15, PMID: 26446607 PMC4702558

[ref3] Bao D. Q. Xue R. X. Zhang M. Lu C. Y. Ma T. X. Ren C. C.. (2021). N-linked glycosylation plays an important role in budding of neuraminidase protein and virulence of influenza viruses. J. Virol. 95, e02042–e02020. doi: 10.1128/JVI.02042-20 33177197 PMC7925095

[ref4] Bergmann S. Elbahesh H. (2019). Targeting the proviral host kinase, FAK, limits influenza a virus pathogenesis and NFkB-regulated pro-inflammatory responses. Virology 534, 54–63. doi: 10.1016/j.virol.2019.05.020 31176924

[ref5] Chang D. Zaia J. (2019). Why glycosylation matters in building a better flu vaccine*. Mol. Cell. Proteomics 18, 2348–2358. doi: 10.1074/mcp.R119.001491 31604803 PMC6885707

[ref6] Chen W. Calvo P. A. Malide D. Gibbs J. Schubert U. Bacik I.. (2001). A novel influenza a virus mitochondrial protein that induces cell death. Nat. Med. 7, 1306–1312. doi: 10.1038/nm1201-1306, PMID: 11726970

[ref7] Chen X. F. He Y. F. Zhu Y. L. Du J. Sun H. X. (2021). Linc-AAM facilitates gene expression contributing to macrophage activation and adaptive immune responses. Cell Rep. 5:108584. doi: 10.1016/j.celrep.2020.108584 33406422

[ref8] Chen Y. Lei X. Jiang Z. Fitzgerald K. A. (2021). Cellular nucleic acid-binding protein is essential for type I interferon-mediated immunity to RNA virus infection. Proc. Natl. Acad. Sci. USA 118:e2100383118. doi: 10.1073/pnas.2100383118, PMID: 34168080 PMC8255963

[ref9] Chen L. Xing C. Ma G. Luo J. Su W. Li M.. (2018). N-myc downstream-regulated gene 1 facilitates influenza a virus replication by suppressing canonical NF-kappaB signaling. Virus Res. 252, 22–28. doi: 10.1016/j.virusres.2018.05.001, PMID: 29730307

[ref10] Daniels R. Kurowski B. Johnson A. E. Hebert D. N. (2003). N-linked glycans direct the cotranslational folding pathway of hemagglutinin. Mol. Cell 11, 79–90. doi: 10.1016/S1097-2765(02)00821-3, PMID: 12535523

[ref11] De Jong J. C. Beyer W. E. Palache A. M. Rimmelzwaan G. F. Osterhaus A. D. (2000). Mismatch between the 1997/1998 influenza vaccine and the major epidemic a (H3N2) virus strain as the cause of an inadequate vaccine-induced antibody response to this strain in the elderly. J. Med. Virol. 61, 94–99. doi: 10.1002/(SICI)1096-9071(200005)61:1<94::AID-JMV15>3.0.CO;2-C, PMID: 10745239

[ref12] Du Y. Yang F. Wang Q. Xu N. Xie Y. Chen S.. (2020). Influenza a virus antagonizes type I and type II interferon responses via SOCS1-dependent ubiquitination and degradation of JAK1. Virol. J. 17:74. doi: 10.1186/s12985-020-01348-4 32532301 PMC7291424

[ref13] Ekiert D. C. Friesen R. H. E. Bhabha G. Kwaks T. Jongeneelen M. Yu W. L.. (2011). A highly conserved neutralizing epitope on group 2 influenza a viruses. Science 333, 843–850. doi: 10.1126/science.1204839, PMID: 21737702 PMC3210727

[ref14] Feng M. K. Zhang Q. Wu W. J. Chen L. Z. Gu S. Y. Ye Y. L.. (2021). Inducible guanylate-binding protein 7 facilitates influenza a virus replication by suppressing innate immunity via NF-κB and JAK-STAT signaling pathways. J. Virol. 95, e02038–e02020. doi: 10.1128/JVI.02038-20 33408175 PMC8094947

[ref15] Fereidouni S. Starick E. Karamendin K. Genova C. D. Scott S. D. Khan Y.. (2023). Genetic characterization of a new candidate hemagglutinin subtype of influenza a viruses. Emerg. Microb. Infect. 12:2225645. doi: 10.1080/22221751.2023.2225645 PMC10308872 37335000

[ref16] Gack M. U. Albrecht R. A. Urano T. Inn K. S. Huang I. C. Carnero E.. (2009). Influenza a virus NS1 targets the ubiquitin ligase TRIM25 to evade recognition by the host viral RNA sensor RIG-I. Cell Host Microbe 5, 439–449. doi: 10.1016/j.chom.2009.04.006 19454348 PMC2737813

[ref17] Gao R. Cao B. Hu Y. Feng Z. Wang D. Hu W.. (2013). Human infection with a novel avian-origin influenza a (H7N9) virus. N. Engl. J. Med. 368, 1888–1897. doi: 10.1056/NEJMoa1304459, PMID: 23577628

[ref18] Ge P. Ross T. M. (2021). Evolution of a (H1N1) pdm09 influenza virus masking by glycosylation. Expert Rev. Vaccines 20, 519–526. doi: 10.1080/14760584.2021.1908135, PMID: 33756084

[ref19] Gopal R. Marinelli M. A. Alcorn J. F. (2020). Immune mechanisms in cardiovascular diseases associated with viral infection. Front. Immunol. 11:570681. doi: 10.3389/fimmu.2020.570681 33193350 PMC7642610

[ref20] Graef K. M. Vreede F. T. Lau Y. F. McCall A. W. Carr S. M. Subbarao K.. (2010). The PB2 subunit of the influenza virus RNA polymerase affects virulence by interacting with the mitochondrial antiviral signaling protein and inhibiting expression of beta interferon. J. Virol. 84, 8433–8445. doi: 10.1128/JVI.00879-10, PMID: 20538852 PMC2919034

[ref21] Graham M. B. Braciale V. L. Braciale T. J. (1994). Influenza virus-specific CD4+ T helper type 2 T lymphocytes do not promote recovery from experimental virus infection. J. Exp. Med. 180, 1273–1282. doi: 10.1084/jem.180.4.1273, PMID: 7931062 PMC2191682

[ref22] Hensley S. E. Das S. R. Gibbs J. S. Bailey A. L. Schmidt L. M. Bennink J. R.. (2011). Influenza a virus hemagglutinin antibody escape promotes neuraminidase antigenic variation and drug resistance. PLoS One 6:e15190. doi: 10.1371/journal.pone.0015190 21364978 PMC3043005

[ref23] Hervé P. L. Lorin V. Jouvion G. Da Costa B. Escriou N. (2015). Addition of N-glycosylation sites on the globular head of the H5 hemagglutinin induces the escape of highly pathogenic avian influenza a H5N1 viruses from vaccine-induced immunity. Virology 486, 134–145. doi: 10.1016/j.virol.2015.08.033, PMID: 26433051

[ref24] Ho B. C. Yu I. S. Lu L. F. Rudensky A. Chen H. Y. Tsai C. W.. (2014). Inhibition of miR-146a prevents enterovirus-induced death by restoring the production of type I interferon. Nat. Commun. 5:3344. doi: 10.1038/ncomms4344 24561744

[ref25] Hu J. Kong M. Cui Z. Gao Z. Ma C. Hu Z.. (2020). PA-X protein of H5N1 avian influenza virus inhibits NF-kappaB activity, a potential mechanism for PA-X counteracting the host innate immune responses. Vet. Microbiol. 250:108838. doi: 10.1016/j.vetmic.2020.108838 33045633

[ref26] Hu Y. Pan Q. Zhou K. Ling Y. Wang H. Li Y. (2022). RUNX1 inhibits the antiviral immune response against influenza a virus through attenuating type I interferon signaling. Virol. J. 19:39. doi: 10.1186/s12985-022-01764-8 35248104 PMC8897766

[ref27] Huang M. T. Zhang S. Wu Y. N. Li W. Li Y. C. Zhou C. S.. (2021). Dual R108K and G189D mutations in the NS1 protein of a/H1N1 influenza virus counteract host innate immune responses. Viruses 13:905. doi: 10.3390/v13050905, PMID: 34068322 PMC8153306

[ref28] Iwasaki A. Medzhitov R. (2015). Control of adaptive immunity by the innate immune system. Nat. Immunol. 16, 343–353. doi: 10.1038/ni.3123, PMID: 25789684 PMC4507498

[ref29] Jagger B. W. Wise H. M. Kash J. C. Walters K. A. Wills N. M. Xiao Y. L.. (2012). An overlapping protein-coding region in influenza a virus segment 3 modulates the host response. Science 337, 199–204. doi: 10.1126/science.1222213 22745253 PMC3552242

[ref30] Jiang J. Li Y. Sun Z. Gong L. Li X. Shi F.. (2022). LncNSPL facilitates influenza a viral immune escape by restricting TRIM25-mediated K63-linked RIG-I ubiquitination. iScience. 25:104607. doi: 10.1016/j.isci.2022.104607 35800772 PMC9253711

[ref31] Jin F. Dong X. M. Wan Z. M. Ren D. Liu M. Geng T. Y.. (2019). A single mutation N166D in hemagglutinin affects antigenicity and pathogenesis of H9N2 avian influenza virus. Viruses-Basel 11:709. doi: 10.3390/v11080709, PMID: 31382442 PMC6723300

[ref32] Kaverin N. V. Rudneva I. A. Ilyushina N. A. Lipatov A. S. Krauss S. Webster R. G. (2004). Structural differences among hemagglutinins of influenza a virus subtypes are reflected in their antigenic architecture: analysis of H9 escape mutants. J. Virol. 78, 240–249. doi: 10.1128/JVI.78.1.240-249.2004, PMID: 14671105 PMC303415

[ref33] Kosik I. Ince W. L. Gentles L. E. Oler A. J. Kosikova M. Angel M.. (2018). Influenza a virus hemagglutinin glycosylation compensates for antibody escape fitness costs. PLoS Pathog. 14:e1006796. doi: 10.1371/journal.ppat.1006796, PMID: 29346435 PMC5773227

[ref34] Krammer F. (2019). The human antibody response to influenza a virus infection and vaccination. Nat. Rev. Immunol. 19, 383–397. doi: 10.1038/s41577-019-0143-6, PMID: 30837674

[ref35] Krammer F. Smith G. J. D. Fouchier R. A. M. Peiris M. Kedzierska K. Doherty P. C.. (2018). Influenza. Nat. Rev. Dis. Primers 4:3. doi: 10.1038/s41572-018-0002-y 29955068 PMC7097467

[ref36] Kumar K. G. Barriere H. Carbone C. J. Liu J. Swaminathan G. Xu P.. (2007). Site-specific ubiquitination exposes a linear motif to promote interferon-alpha receptor endocytosis. J. Cell Biol. 179, 935–950. doi: 10.1083/jcb.200706034 18056411 PMC2099190

[ref37] Kumar K. G. Krolewski J. J. Fuchs S. Y. (2004). Phosphorylation and specific ubiquitin acceptor sites are required for ubiquitination and degradation of the IFNAR1 subunit of type I interferon receptor. J. Biol. Chem. 279, 46614–46620. doi: 10.1074/jbc.M407082200, PMID: 15337770

[ref38] Kumari R. Guo Z. Kumar A. Wiens M. Gangappa S. Katz J. M.. (2020). Influenza virus NS1-C/EBPbeta gene regulatory complex inhibits RIG-I transcription. Antivir. Res. 176:104747. doi: 10.1016/j.antiviral.2020.104747, PMID: 32092305 PMC10773002

[ref39] Li X. Guo G. Lu M. Chai W. Li Y. Tong X.. (2019). Long noncoding RNA Lnc-MxA inhibits Beta interferon transcription by forming RNA-DNA triplexes at its promoter. J. Virol. 93:e00786–19. doi: 10.1128/JVI.00786-19, PMID: 31434735 PMC6803265

[ref40] Li M. Qi W. Chang Q. Chen R. Zhen D. Liao M.. (2021). Influenza a virus protein PA-X suppresses host Ankrd17-mediated immune responses. Microbiol. Immunol. 65, 48–59. doi: 10.1111/1348-0421.12863 33241870

[ref41] Liedmann S. Hrincius E. R. Guy C. Anhlan D. Dierkes R. Carter R.. (2014). Viral suppressors of the RIG-I-mediated interferon response are pre-packaged in influenza virions. Nat. Commun. 5:5645. doi: 10.1038/ncomms6645 25487526 PMC4268707

[ref42] Lin C. Y. Shih M. C. Chang H. C. Lin K. J. Chen L. F. Huang S. W.. (2021). Influenza a virus NS1 resembles a TRAF3-interacting motif to target the RNA sensing-TRAF3-type I IFN axis and impair antiviral innate immunity. J. Biomed. Sci. 28:66. doi: 10.1186/s12929-021-00764-0 34610835 PMC8491413

[ref43] Liu Y. C. Mok B. W. Wang P. Kuo R. L. Chen H. Shih S. R. (2021). Cellular 5′-3' mRNA exoribonuclease XRN1 inhibits interferon Beta activation and facilitates influenza a virus replication. MBio 12:e0094521. doi: 10.1128/mBio.00945-21, PMID: 34311580 PMC8406323

[ref44] Lyashko A. V. Timofeeva T. A. Rudneva I. A. Lomakina N. F. Treshchalina A. A. Gambaryan A. S.. (2024). Antigenic architecture of the H7N2 influenza virus hemagglutinin belonging to the north American lineage. Int. J. Mol. Sci. 25:212. doi: 10.3390/ijms25010212 PMC10779424 38203384

[ref45] Mellman I. Steinman R. M. (2001). Dendritic cells: specialized and regulated antigen processing machines. Cell 106, 255–258. doi: 10.1016/S0092-8674(01)00449-4, PMID: 11509172

[ref46] Moeller A. Kirchdoerfer R. N. Potter C. S. Carragher B. Wilson I. A. (2012). Organization of the influenza virus replication machinery. Science 338, 1631–1634. doi: 10.1126/science.1227270, PMID: 23180774 PMC3578580

[ref47] Moran T. M. Park H. Fernandez-Sesma A. Schulman J. L. (1999). Th2 responses to inactivated influenza virus can be converted to Th1 responses and facilitate recovery from heterosubtypic virus infection. J. Infect. Dis. 180, 579–585. doi: 10.1086/314952, PMID: 10438342

[ref48] Negishi H. Taniguchi T. Yanai H. (2018). The interferon (IFN) class of cytokines and the IFN regulatory factor (IRF) transcription factor family. Cold Spring Harb. Perspect. Biol. 10:a028423. doi: 10.1101/cshperspect.a028423, PMID: 28963109 PMC6211389

[ref49] Okamatsu M. Sakoda Y. Kishida N. Isoda N. Kida H. (2008). Antigenic structure of the hemagglutinin of H9N2 influenza viruses. Arch. Virol. 153, 2189–2195. doi: 10.1007/s00705-008-0243-2 18989614 PMC7087127

[ref50] Olsen B. Munster V. J. Wallensten A. Waldenstrom J. Osterhaus A. D. Fouchier R. A. (2006). Global patterns of influenza a virus in wild birds. Science 312, 384–388. doi: 10.1126/science.1122438, PMID: 16627734

[ref51] Ouyang W. Cen M. Y. Yang L. P. Zhang W. Y. Xia J. Y. Xu F. (2021). NMI facilitates influenza a virus infection by promoting degradation of IRF7 through TRIM21. Am. J. Respir. Cell Mol. Biol. 65, 30–40. doi: 10.1165/rcmb.2020-0391OC 33761305

[ref52] Park H. S. Lu Y. Pandey K. Liu G. Zhou Y. (2021). NLRP3 Inflammasome activation enhanced by TRIM25 is targeted by the NS1 protein of 2009 pandemic influenza a virus. Front. Microbiol. 12:778950. doi: 10.3389/fmicb.2021.778950 34867921 PMC8633893

[ref53] Peacock T. Reddy K. James J. Adamiak B. Barclay W. Shelton H.. (2016). Antigenic mapping of an H9N2 avian influenza virus reveals two discrete antigenic sites and a novel mechanism of immune escape. Sci. Rep. 7:18745. doi: 10.1038/srep18745 PMC4704030 26738561

[ref54] Pinar A. Dowling J. K. Bitto N. J. Robertson A. A. Latz E. Stewart C. R.. (2017). PB1-F2 peptide derived from avian influenza a virus H7N9 induces inflammation via activation of the NLRP3 Inflammasome. J. Biol. Chem. 292, 826–836. doi: 10.1074/jbc.M116.756379, PMID: 27913620 PMC5247656

[ref55] Ping J. H. Li C. J. Deng G. H. Jiang Y. P. Tian G. B. Zhang S. X.. (2008). Single-amino-acid mutation in the HA alters the recognition of H9N2 influenza virus by a monoclonal antibody. Biochem. Biophys. Res. Commun. 371, 168–171. doi: 10.1016/j.bbrc.2008.04.045 18424263

[ref56] Qin T. Chen Y. Huangfu D. Miao X. Yin Y. Yin Y.. (2023). PA-X protein of H9N2 subtype avian influenza virus suppresses the innate immunity of chicken bone marrow-derived dendritic cells. Poult. Sci. 102:102304. doi: 10.1016/j.psj.2022.102304, PMID: 36436371 PMC9700306

[ref57] Qin T. Chen Y. Huangfu D. Yin Y. Miao X. Yin Y.. (2022). PA-X protein assists H9N2 subtype avian influenza virus in escaping immune response of mucosal dendritic cells. Transbound. Emerg. Dis. 69, e3088–e3100. doi: 10.1111/tbed.14665, PMID: 35855630

[ref58] Rashid M. U. Gao A. Coombs K. M. (2022). Influenza a virus uses PSMA2 for downregulation of the NRF2-mediated oxidative stress response. J. Virol. 96:e0199021. doi: 10.1128/jvi.01990-21 35019712 PMC8906419

[ref59] Richard M. Fouchier R. A. (2016). Influenza a virus transmission via respiratory aerosols or droplets as it relates to pandemic potential. FEMS Microbiol. Rev. 40, 68–85. doi: 10.1093/femsre/fuv039 26385895 PMC5006288

[ref60] Rigby R. E. Wise H. M. Smith N. Digard P. Rehwinkel J. (2019). PA-X antagonises MAVS-dependent accumulation of early type I interferon messenger RNAs during influenza a virus infection. Sci. Rep. 9:7216. doi: 10.1038/s41598-019-43632-6 31076606 PMC6510759

[ref61] Schaap I. A. Eghiaian F. (2012). Effect of envelope proteins on the mechanical properties of influenza virus. J. Biol. Chem. 287, 41078–41088. doi: 10.1074/jbc.M112.412726, PMID: des Georges A, Veigel C 23048030 PMC3510809

[ref62] Sealy J. E. Peacock T. P. Sadeyen J. R. Chang P. X. Everest H. J. Bhat S.. (2020). Adsorptive mutation and N-linked glycosylation modulate influenza virus antigenicity and fitness. Emerg. Microb. Infect. 9, 2622–2631. doi: 10.1080/22221751.2020.1850180, PMID: 33179567 PMC7738305

[ref63] Shang J. Zheng Y. Mo J. Wang W. Luo Z. Li Y.. (2021). Sox4 represses host innate immunity to facilitate pathogen infection by hijacking the TLR signaling networks. Virulence 12, 704–722. doi: 10.1080/21505594.2021.1882775 33517839 PMC7894441

[ref64] Shi J. Feng P. Gu T. (2020). MicroRNA-21-3p modulates FGF2 to facilitate influenza a virus H5N1 replication by refraining type I interferon response. Biosci. Rep. 40:BSR20200158. doi: 10.1042/bsr20200158 32432671 PMC7256676

[ref65] Shin D. Lee J. Park J. H. Min J. Y. (2017). Double plant homeodomain fingers 2 (DPF2) promotes the immune escape of influenza virus by suppressing Beta interferon production. J. Virol. 91, e02260–e02216. doi: 10.1128/JVI.02260-16 28404846 PMC5446637

[ref66] Sun X. Feng W. Guo Y. Wang Q. Dong C. Zhang M.. (2018). MCPIP1 attenuates the innate immune response to influenza a virus by suppressing RIG-I expression in lung epithelial cells. J. Med. Virol. 90, 204–211. doi: 10.1002/jmv.24944 28892164

[ref67] Tan X. Sun L. Chen J. Chen Z. J. (2018). Detection of microbial infections through innate immune sensing of nucleic acids. Ann. Rev. Microbiol. 72, 447–478. doi: 10.1146/annurev-micro-102215-095605 30200854

[ref68] Thornlow D. N. Macintyre A. N. Oguin T. H. Karlsson A. B. Stover E. L. Lynch H. E.. (2021). Altering the immunogenicity of hemagglutinin immunogens by Hyperglycosylation and disulfide stabilization. Front. Immunol. 7:737973. doi: 10.3389/fimmu.2021.737973 PMC8528956 34691043

[ref69] To E. E. Vlahos R. Luong R. Halls M. L. Reading P. C. King P. T.. (2017). Endosomal NOX2 oxidase exacerbates virus pathogenicity and is a target for antiviral therapy. Nat. Commun. 8:69. doi: 10.1038/s41467-017-00057-x 28701733 PMC5507984

[ref70] Wagner R. Wolff T. Herwig A. Pleschka S. Klenk H. D. (2000). Interdependence of hemagglutinin glycosylation and neuraminidase as regulators of influenza virus growth: a study by reverse genetics. J. Virol. 74, 6316–6323. doi: 10.1128/JVI.74.14.6316-6323.2000, PMID: 10864641 PMC112137

[ref71] Wan Z. M. Ye J. Q. Xu L. L. Shao H. X. Jin W. J. Qian K.. (2014). Antigenic mapping of the hemagglutinin of an H9N2 avian influenza virus reveals novel critical amino acid positions in antigenic sites. J. Virol. 88, 3898–3901. doi: 10.1128/JVI.03440-13, PMID: 24429369 PMC3993533

[ref72] Wang J. Cen S. (2020). Roles of lncRNAs in influenza virus infection. Emerg. Microb. Infect. 9, 1407–1414. doi: 10.1080/22221751.2020.1778429, PMID: 32543285 PMC7473136

[ref73] Wang S. Dai T. Qin Z. R. Pan T. Chu F. Lou L. F.. (2021). Targeting liquid-liquid phase separation of SARS-CoV-2 nucleocapsid protein promotes innate antiviral immunity by elevating MAVS activity. Nat. Cell Biol. 23, 718–732. doi: 10.1038/s41556-021-00710-0, PMID: 34239064

[ref74] Wang Q. S. Pan W. L. Wang S. Pan C. Ning H. Y. Huang S. L.. (2021). Protein tyrosine phosphatase SHP2 suppresses host innate immunity against influenza a virus by regulating EGFR-mediated signaling. J. Virol. 95, e02001–e02020. doi: 10.1128/JVI.02001-20 33361428 PMC8094946

[ref75] Wang T. Wei F. Jiang Z. Song J. Li C. Liu J. (2022). Influenza virus NS1 interacts with 14-3-3epsilon to antagonize the production of RIG-I-mediated type I interferons. Virology 574, 47–56. doi: 10.1016/j.virol.2022.07.002 35926243

[ref76] Wang R. Zhu Y. Ren C. Yang S. Tian S. Chen H.. (2021). Influenza a virus protein PB1-F2 impairs innate immunity by inducing mitophagy. Autophagy 17, 496–511. doi: 10.1080/15548627.2020.1725375, PMID: 32013669 PMC8007153

[ref77] Watanabe T. Watanabe S. Maher E. A. Neumann G. Kawaoka Y. (2014). Pandemic potential of avian influenza a (H7N9) viruses. Trends Microbiol. 22, 623–631. doi: 10.1016/j.tim.2014.08.008, PMID: 25264312 PMC4252989

[ref78] Webby R. J. Webster R. G. (2001). Emergence of influenza a viruses. Philos. Trans. R. Soc. Lond. Ser. B Biol. Sci. 356, 1817–1828. doi: 10.1098/rstb.2001.0997 11779380 PMC1088557

[ref79] Wolf J. J. Xia C. Studstill C. J. Ngo H. Brody S. L. Anderson P. E.. (2021). Influenza a virus NS1 induces degradation of sphingosine 1-phosphate lyase to obstruct the host innate immune response. Virology 558, 67–75. doi: 10.1016/j.virol.2021.02.006, PMID: 33730651 PMC8109848

[ref80] Xia C. Vijayan M. Pritzl C. J. Fuchs S. Y. McDermott A. B. Hahm B. (2015). Hemagglutinin of influenza a virus antagonizes type I interferon (IFN) responses by inducing degradation of type I IFN receptor 1. J. Virol. 90, 2403–2417. doi: 10.1128/JVI.02749-15, PMID: 26676772 PMC4810695

[ref81] Xia C. Wolf J. J. Sun C. Xu M. Studstill C. J. Chen J.. (2020). PARP1 enhances influenza a virus propagation by facilitating degradation of host type I interferon receptor. J. Virol. 94, e01572–e01519. doi: 10.1128/JVI.01572-19 PMC7081902 31915279

[ref82] Xia C. Wolf J. J. Vijayan M. Studstill C. J. Ma W. Hahm B. (2018). Casein kinase 1alpha mediates the degradation of receptors for type I and type II interferons caused by hemagglutinin of influenza a virus. J. Virol. 92, e00006–e00018. doi: 10.1128/JVI.00006-18 29343571 PMC5972889

[ref83] Xiao Y. Evseev D. Stevens C. A. Moghrabi A. Miranzo-Navarro D. Fleming-Canepa X.. (2020). Influenza PB1-F2 inhibits avian MAVS signaling. Viruses 12:409. doi: 10.3390/v12040409 32272772 PMC7232376

[ref84] Xu M. Xia S. Wang M. Liu X. Li X. Chen W.. (2022). Enzymatic independent role of sphingosine kinase 2 in regulating the expression of type I interferon during influenza a virus infection. PLoS Pathog. 18:e1010794. doi: 10.1371/journal.ppat.1010794, PMID: 36070294 PMC9451060

[ref85] Xu C. Z. Zhang N. X. Yang Y. Y. Liang W. H. Zhang Y. P. Wang J. F.. (2022). Immune escape adaptive mutations in hemagglutinin are responsible for the antigenic drift of Eurasian avian-like H1N1 swine influenza viruses. J. Virol. 96:e0097122. doi: 10.1128/jvi.00971-22 35916512 PMC9400474

[ref86] Yang W. Schountz T. Ma W. (2021). Bat influenza viruses: current status and perspective. Viruses 13:547. doi: 10.3390/v13040547 33805956 PMC8064322

[ref87] Yao J. Lin C. H. Jiang J. J. Zhang X. J. Li F. X. Liu T. X.. (2021). lncRNA-HEIM facilitated liver fibrosis by up-regulating TGF-β expression in long-term outcome of chronic hepatitis B. Front. Immunol. 8:666370. doi: 10.3389/fimmu.2021.666370 PMC8217658 34168644

[ref88] Yi C. Zhao Z. Wang S. Sun X. Zhang D. Sun X.. (2017). Influenza a virus PA antagonizes interferon-beta by interacting with interferon regulatory factor 3. Front. Immunol. 8:1051. doi: 10.3389/fimmu.2017.01051 28955326 PMC5600993

[ref89] Yu M. Guo Y. Zhao L. Lu Y. Liu Q. Li Y.. (2022). D2I and F9Y mutations in the NS1 protein of influenza a virus affect viral replication via regulating host innate immune responses. Viruses 14:1206. doi: 10.3390/v14061206, PMID: 35746676 PMC9228823

[ref90] Zeng Y. Xu S. Wei Y. Zhang X. Wang Q. Jia Y.. (2021). The PB1 protein of influenza a virus inhibits the innate immune response by targeting MAVS for NBR1-mediated selective autophagic degradation. PLoS Pathog. 17:e1009300. doi: 10.1371/journal.ppat.1009300 33577621 PMC7880438

[ref91] Zhang F. Lin X. Yang X. Lu G. Zhang Q. Zhang C. (2019). MicroRNA-132-3p suppresses type I IFN response through targeting IRF1 to facilitate H1N1 influenza a virus infection. Biosci. Rep. 39:BSR20192769. doi: 10.1042/bsr20192769 31746331 PMC6904772

[ref92] Zhang B. Liu M. Huang J. Zeng Q. Zhu Q. Xu S.. (2022). H1N1 influenza a virus protein NS2 inhibits innate immune response by targeting IRF7. Viruses 14:2411. doi: 10.3390/v14112411, PMID: 36366509 PMC9694023

[ref93] Zhang N. Ma Y. Tian Y. Zhou Y. Tang Y. Hu S. (2021). Downregulation of microRNA-221 facilitates H1N1 influenza a virus replication through suppression of type-IFN response by targeting the SOCS1/NF-κB pathway. Mol. Med. Rep. 24:497. doi: 10.3892/mmr.2021.12136 33955508 PMC8127060

[ref94] Zhang B. Xu S. Liu M. Wei Y. Wang Q. Shen W.. (2023). The nucleoprotein of influenza a virus inhibits the innate immune response by inducing mitophagy. Autophagy 19, 1916–1933. doi: 10.1080/15548627.2022.2162798 36588386 PMC10283423

[ref95] Zhang Q. Zhang X. Lei X. Wang H. Jiang J. Wang Y.. (2022). Influenza a virus NS1 protein hijacks YAP/TAZ to suppress TLR3-mediated innate immune response. PLoS Pathog. 18:e1010505. doi: 10.1371/journal.ppat.1010505 35503798 PMC9122210

[ref96] Zhao Y. Huang F. Zou Z. Bi Y. Yang Y. Zhang C.. (2022). Avian influenza viruses suppress innate immunity by inducing trans-transcriptional readthrough via SSU72. Cell. Mol. Immunol. 19, 702–714. doi: 10.1038/s41423-022-00843-8 35332300 PMC9151799

[ref97] Zheng H. Qian J. Baker D. P. Fuchs S. Y. (2011). Tyrosine phosphorylation of protein kinase D2 mediates ligand-inducible elimination of the type 1 interferon receptor. J. Biol. Chem. 286, 35733–35741. doi: 10.1074/jbc.M111.263608 21865166 PMC3195636

[ref98] Zheng H. Qian J. Varghese B. Baker D. P. Fuchs S. (2011). Ligand-stimulated downregulation of the alpha interferon receptor: role of protein kinase D2. Mol. Cell. Biol. 31, 710–720. doi: 10.1128/MCB.01154-10 21173164 PMC3028644

[ref99] Zhu R. Xu S. S. Sun W. Y. J. Li Q. Wang S. F. Shi H. Y.. (2022). HA gene amino acid mutations contribute to antigenic variation and immune escape of H9N2 influenza virus. Vet. Res. 53:43. doi: 10.1186/s13567-022-01131-z 35706014 PMC9202205

[ref100] Zhu Y. B. Yang D. Ren Q. Yang Y. Liu X. Xu X. L.. (2015). Identification and characterization of a novel antigenic epitope in the hemagglutinin of the escape mutants of H9N2 avian influenza viruses. Vet. Microbiol. 178, 144–149. doi: 10.1016/j.vetmic.2015.04.012 25934533

